# Chronic pneumonia with *Pseudomonas aeruginosa *and impaired alveolar fluid clearance

**DOI:** 10.1186/1465-9921-6-17

**Published:** 2005-02-11

**Authors:** Sophie Boyer, Karine Faure, Florence Ader, Marie Odile Husson, Eric Kipnis, Thierry Prangere, Xavier Leroy, Benoit P Guery

**Affiliations:** 1 Laboratoire de recherche en Pathologie Infectieuse, EA 2689. Faculté de Médecine de Lille, 59031 Lille Cedex, France; 2 Laboratoire de Bactériologie; Hôpital Calmette, CHRU de Lille, Lille, France; 3 Laboratoire de Biophysique, CHRU, Lille, France; 4 Laboratoire d'anatomo-pathologie, CHRU Lille, France

## Abstract

**Background:**

While the functional consequences of acute pulmonary infections are widely documented, few studies focused on chronic pneumonia. We evaluated the consequences of chronic *Pseudomonas *lung infection on alveolar function.

**Methods:**

*P. aeruginosa*, included in agar beads, was instilled intratracheally in Sprague Dawley rats. Analysis was performed from day 2 to 21, a control group received only sterile agar beads. Alveolar-capillary barrier permeability, lung liquid clearance (LLC) and distal alveolar fluid clearance (DAFC) were measured using a vascular (^131^I-Albumin) and an alveolar tracer (^125^I-Albumin).

**Results:**

The increase in permeability and LLC peaked on the second day, to return to baseline on the fifth. DAFC increased independently of TNF-α or endogenous catecholamine production. Despite the persistence of the pathogen within the alveoli, DAFC returned to baseline on the 5^th ^day. Stimulation with terbutaline failed to increase DAFC. Eradication of the pathogen with ceftazidime did not restore DAFC response.

**Conclusions:**

From these results, we observe an adequate initial alveolar response to increased permeability with an increase of DAFC. However, DAFC increase does not persist after the 5^th ^day and remains unresponsive to stimulation. This impairment of DAFC may partly explain the higher susceptibility of chronically infected patients to subsequent lung injury.

## Introduction

*Pseudomonas aeruginosa *is a Gram negative bacteria producing a wide array of virulence factors frequently responsible for chronic airway infections in cystic fibrosis (CF) or chronic obstructive pneumonia disease (COPD) patients, as well as acute nosocomial airway infections in intensive care units [1-3].

In acute *P. aeruginosa *pneumonia, the functional consequences, and particularly lung fluid movements, have been studied extensively. Lung fluid balance is the result of fluid movements following active ion transport by functional alveolar cells, and permeability of the alveolar capillary barrier. In *P. aeruginosa*-induced acute lung injury (ALI), distal airspace fluid clearance (DAFC) is typically increased at 24 hours through a TNF-α pathway [4]. Studies have also shown that the capacity of maintaining alveolar active fluid transport is correlated with patient outcome in ALI [5,6]. Lung liquid clearance (LLC) is another functional marker reflecting the capacity of the lung to evacuate fluid instilled in the alveoli outside the lung, LLC involves DAFC, epithelial and endothelial permeabilities [7]. We previously showed that, even though DAFC is upregulated, LLC is decreased at both 4 and 24 hours in ALI [7] reflecting a major endothelial injury overwhelming the alveolar response.

In chronic infection, these functional consequences on lung fluid balance are less clear. In the 70's, Cash developed an experimental model of chronic pneumonia by intra tracheal injection of *P. aeruginosa *embedded in agar beads [8]. Most of the work performed with this model has focused on immunological, inflammatory, or nutritional aspects [9-12]. To the best of our knowledge, no previous work has tried to evaluate alveolar permeability and lung fluid transport in *P. aeruginosa *chronic lung infection. In order to elucidate these functional aspects we studied lung fluid transport in an experimental model of chronic *P. aeruginosa *lung infection in the rat. After the validation of the experimental model, we studied alveolar function: alveolar-capillary barrier permeability, lung liquid clearance, distal airspace fluid clearance and its pharmacologic stimulation.

## Materials and Methods

### Animals

Specific pathogen-free Sprague Dawley rats (n = 280) (230–270 g), (Depre, St Doulchard, France) were housed in the Lille University Animal Care Facility and allowed food and water *ad lib*. All experiments were performed with approval of the Lille Institutional Animal Care and Use Committee.

### Preparation of the bacterial inoculum

The methodology was adapted from Cash et al [8]. Briefly, *P. aeruginosa *(PAO1 strain) was incubated in 125 ml of tryptic soy broth at 37°C in a rotating shaking water bath for 8 hours. The culture was then washed twice, and resuspended in phosphate-buffered saline. The resulting bacterial suspension was 1 × 10^9 ^CFU/ml. A sample of 1 mL of this suspension was mixed in agarose and mineral oil (Sigma Diagnoses, St Louis, USA) at 56°C. The resulting oil-agar emulsion was cooled to obtain agar beads. Dilutions of the final suspension were cultured to determine the size of the final inoculum.

### Experimental infection

Under a short general anesthesia with ether (Mallinkrodt, Paris, France), with sterile surgical conditions, a small midline incision was made on the neck ventral surface after swabbing it with ethanol. The trachea was exposed by blunt dissection. Using a 28-gauge needle, 0.1 mL of agar beads followed by 0.5 mL of air were inoculated intra-tracheally.

### Quantitative bacteriological analysis

After exsanguination of the animal, the lungs were isolated and homogenized in 2 mL of sterile isotonic saline. Bacterial culture after serial dilutions was performed and bacterial colonies counted after 12 h at 37°C.

### Antimicrobial therapy

In a subgroup of animals, ceftazidime (GlaxoSmithKline, Marly-le-Roi, France), 100 mg/kg, was administered in the peritoneal cavity every 8 hours during 72 hours. Lungs were harvested, homogenized and cultures were performed to confirm bacterial eradication. Serum ceftazidime levels were measured in HPLC.

### Broncho-alveolar lavage (BAL)

Broncho-alveolar lavage (BAL) was performed by cannulating the trachea. Lungs from each experimental group were lavaged with a total of 20 ml in 5-ml aliquots of PBS with EDTA (3 mM). BAL fluid samples were filtered and immediately frozen at -80°C. A cell count was performed directly. Cellular monolayers were prepared with a cytocentrifuge and stained with Wright-Giemsa stain. Cellular morphotype differential was obtained by counting 200 cells/sample and expressing each type of cell as a percentage of the total number counted. Protein concentration in the BAL was measured with an automated analyzer (Hitachi 917, Japan).

### Histological study

After a vascular flushing with sterile isotonic saline through the pulmonary artery, the lungs were removed. Samples were fixed by intratracheal instillation of paraformaldehyde 10 %. Samples were included in paraffin and sections of 5 μm were realized. Analysis was performed after coloration with Hematoxyline-Eosine-Safran (Zeiss, LEO 906).

### Serum and BAL TNF-α measurement

Levels of tumor necrosis factor α (TNF-α), in the serum, and the BAL fluid, were determined by use of commercial immunoassay kits (ELISA) specific for rat cytokines (Quantikine Murine rat TNFα, R&D Systems, Abingdon OX, UK). The reading was performed with a microplate reader Digiscan (Spectracount Packard Instrument Company; Meriden CT USA).

### BAL and serum measurement of epinephrine and nor-epinephrine

Blood and broncho-alveolar lavage fluid were collected on heparin/Na-metabisulfite coated tubes. The samples were centrifuged (2500 g, 4°C), supernatants were frozen (-80°C).

Catecholamines are specifically fixed on alumina (pH = 8.7), the eluent is analyzed with an inversed phase H.P.L.C (Coulochem II ESA). The results are expressed in μg/L.

### Functional study

#### Surgical preparation

Sprague-Dawley male rats were anesthetized with pentobarbital (Sanofi, Libourne, France). A catheter (PE-50) was inserted into the left carotid artery in order to monitor systemic arterial pressure (Acqknowledge Software v 3.7.1, Biopac systems, Santa Barbara, CA, USA) and obtain blood samples. An endotracheal tube (PE-220) was inserted through a tracheostomy. The rats were ventilated with a constant volume pump (Harvard Apparatus, South Natick, MA) with an inspired O_2 _fraction of 1.0, a peak airway pressure of 8–12 cmH_2_O, and a positive end expiratory pressure of 2 cmH_2_O. The animals were placed in left decubitus position until the end of the protocol. The body temperature was maintained at 37°C.

#### Preparation of the instillate

The test solution, used for alveolar instillation, was prepared as follows : briefly, a 5% bovine albumin solution was prepared using Ringer lactate and was adjusted with NaCl to be isoosmolar with the rat circulating plasma [13,14]. A sample of the instilled solution was saved for total protein measurement, and water to dry weight ratio measurements. In different experimental groups, terbutaline (10^-4 ^M) (Sigma Aldrich, St Quentin Fallavier, France) was added to the instillate or injected intra-peritoneally to the animals.

#### General Protocol

For all ventilated rats experiments, the following general protocol was used. After the surgical preparation, heart rate and blood pressure were allowed to stabilize for 1 hour. To calculate the flux of plasma protein into the lung interstitium, a vascular tracer, 1 μCi of ^131^I-labeled human albumin, was injected into the bloodstream [14,15]. ^131^I-HSA was prepared in our institution according to a standardized technique. Administration of the instillate (3 ml/kg) was performed into the left lung over a 2-min period, using a 1-ml syringe and polypropylene tube (PE 50, Intramedic, Becton Dickinson, Sparks, MD, USA)[13].

One hour after the beginning of the alveolar instillation, the rat was exanguinated. The lungs were removed, and fluid from the distal airspaces was obtained (aspirate). The total protein concentration and the radioactivity of the liquid sampled were measured. Right and left lungs were homogenized separately for water to dry weight ratio measurements and radioactivity counts.

#### Measurements

• Hemodynamics, pulmonary gas exchange, and protein concentration

Systemic arterial pressure and airway pressures were measured continuously. Arterial blood gases were measured at one hour intervals. The arterial PO_2 _was used to quantify the oxygenation deficit [13,14]. Samples from instillated protein solution, final distal airspace fluid, and from initial and final blood were collected to measure total protein concentration with an automated analyzer (Hitachi 917, Japan).

• Albumin flux across endothelial and epithelial barriers

The flux of albumin across the lung endothelial and epithelial barriers was used to evaluate the permeability. This method requires measurement of the vascular protein tracer, ^131^I-albumin, in the alveolar and extravascular spaces of the lungs. Endothelial permeability was assessed by measuring the ratio of ^131^-iodine radioactivity in the aspirate to the radioactivity obtained in the plasma (Asp/plasma), it reflects the leak of the vascular tracer in the alveolar compartment. We estimated the quantity of plasma that entered the instilled lungs by measuring the transfer of the vascular protein tracer, ^131^I-albumin, into the extravascular spaces of the instilled lung using the equation of plasma equivalents previously described [7,13,14].

• Extravascular lung water (EVLW) and lung liquid clearance (LLC)

The EVLW was estimated by gravimetry: 300 μL of the lung homogenate were weighed, to determine the wet weight, and dessicated at 45°C during 7 days, to obtain the dry weight. The blood fraction was calculated from the homogenate hemoglobin supernatant content. The wet to dry weight ratio (W/D) was estimated using the values of the right lung which was not instilled [7,14,16]. Lung liquid clearance was calculated as previously described [7].

• Distal Airspace Fluid Clearance (DAFC):

A change of native bovine albumin concentration over the study period (1 h) was used to measure alveolar fluid movement. DAFC was calculated from the ratio of the final unlabeled alveolar protein concentration, compared to the initial instilled alveolar protein concentration.

### Experimental groups

15 experimental groups were constituted for the study:

- A control group (Ctr), which received an intratracheal instillation of sterile saline at the beginning of the protocol

- 7 Sterile groups (St) received an intratracheal instillation of sterile beads and were studied at different days after inoculation: St 1, St 2, St 5, St 8, St 15, St 21 and St 28.

- 7 Pneumonic groups (Pn) received an intratracheal instillation of *Pseudomonas *containing beads and were studied at different days after inoculation: Pn 1, Pn 2, Pn 5, Pn 8, Pn 15, Pn 21, Pn 28.

### Statistical analysis

Comparisons between two groups were made using an unpaired, two tailed Student's *t*-test. Comparisons between more than two groups were made using a one way analysis of variance with *post hoc *test for multiple comparisons. A value of p < 0.05 was considered as significant. The data are expressed as means ± SD.

## Results

### *Pseudomonas* beads instillation is associated with the development of a chronic infection

**Figure 1:**
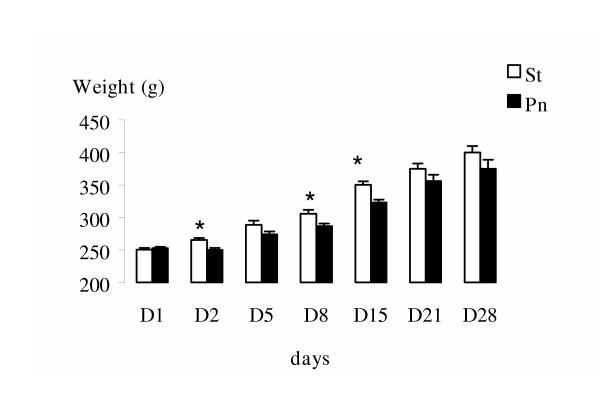
Evolution of animals' weight during the four weeks of the analysis. An initial weight loss is observed for the infected animals compared to the sterile beads group. *Footnote*: Data are mean (± SD). Comparisons between groups were made using analysis of variance. *p < 0.05 vs the Pn group. **Pn**: Pneumonic animals, **St**: animals which received only sterile beads

Clinically, a major weight loss was observed from the second day in *P. aeruginosa *beads infected animals compared to the sterile beads groups (Figure 1). 5% of the infected animals died within the first 48 hours after inoculation, none did in the sterile groups.

Prior to the instillation, the size of the inoculum was 7.9 10^5 ^± 1.5 10^5 ^CFU/mL. Lung bacterial load reached a peak on the second day of the infection; from the 5^th ^day, a progressive decrease occurred to finally remain steady between the 15^th ^day (8.25 ± 5.2 10^4 ^CFU/mL) and the 3^rd ^week (1.67 ± 1.63 10^5 ^CFU/mL).

Total broncho-alveolar lavage (BAL) cells slightly increased in the sterile beads group, the difference was however not statistically significant compared to the control group, the analysis showed that the number of cells peaked on the second day and was constituted, at that time, of 25% polymorphonuclear cells and 75% macrophages. The results were not statistically different over time and therefore pooled in Table 1. In the infected groups, alveolar cellularity was maximum on the 2^nd ^day mostly polymorphonuclear's neutrophils (PMN). From the 8^th ^day, the relative number of PMN progressively decreased as alveolar macrophages increased. All the results are summarized in Table 1.

**Table 1:** Analysis of the bronchoalveolar lavage All the animals who received sterile beads were included in the sterile group and compared to the control and pneumonic groups at respectively 2, 5, 8, 15 and 21 days post instillation.

	Total cells (× 10^6^)/mL	PMNs (%)	Macrophages (%)
Ctr	0.4 ± 0.1	0.5 ± 0.4	98.5 ± 0.5
***St***	3.2 ± 0.6	5.6 ± 4.4	92.9 ± 4.4
***Pn 2***	10.5 ± 2.9*	79.8 ± 5.2*	19.0 ± 4.6*
***Pn 5***	7.9 ± 1.7*	19.0 ± 8.0	79.3 ± 8.4
***Pn 8***	4.9 ± 1.0	3.8 ± 0.7	95.5 ± 1.0
***Pn 15***	4.0 ± 0.8	1.2 ± 0.6	98.8 ± 0.6
***Pn 21***	4.9 ± 1.8	2.0 ± 0.6	97.2 ± 0.6

*Footnote*: Data are mean (± SD). Comparisons between groups were made using analysis of variance with *post hoc *for multiple comparisons. *p < 0.05 vs the other groups.

**PMNs**: Polymorphonuclear neutrophils, **Ctr**: Control group, **St**: Sterile group, **Pn**: Pneumonic group

**Figure 2:**
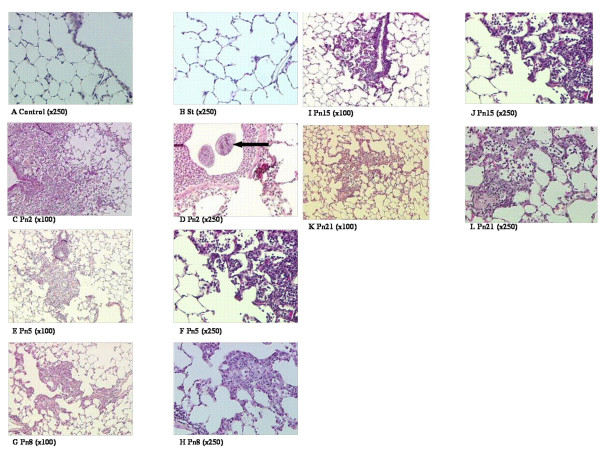
Histological analysis of the different groups, controls and sterile beads instilled animals are compared to pneumonic rats from the second to the 21^st ^day post instillation. Coloration was performed with Hematoxyline-Eosine-Safran. **A**: Control group; **B**: Sterile beads; **C-D**: Pneumonia on the 2^nd ^day (the arrow on panel D underlines infected beads); **E-F**: Pneumonia on the 5^th ^day; **G-H**: Pneumonia on the 8^th ^day; I-J: Pneumonia on the 15^th ^day; **K-L**: Pneumonia on the 21^st ^day.

Histologically, in the infected groups, from the 2^nd ^day, large numbers of PMNs were observed, mostly centered on the alveoli (Figure 2C–D). Agar beads were clearly observed in the Pn2 group (Figure 2D). With time, increased extracellular material became more prominent (Figure 2G–L). The lung architecture of animals inoculated with sterile beads remained strictly normal (Figure 2A–B).

### A transient increase of alveolar-capillary barrier permeability is observed on the second day post infection

No variation in permeability or clearance was observed between St groups, so all the results were included in a single group (St) for the analysis (at least 5 animals were included in each time point). Alveolar-capillary barrier permeability, evaluated by the leakage of the vascular marker into the alveoli (Asp/plasma ratio), was increased in infected animals on the second day compared to the control group (0.59 ± 0.08 vs 0.11 ± 0.02). This ratio came back to control values from the fifth to the 28^th ^day. In the St group a moderate but significant increase of the Asp/plasma ratio was observed throughout the study (0.31 ± 0.04).

### Both lung liquid clearance and DAFC increased on the 2^nd ^day post infection; DAFC increase is not related to a TNF-α or catecholamine dependent mechanism

• Extra-vascular lung water and Lung liquid clearance (LLC)

As shown in Table 2, no difference in wet to dry lung weight ratio was observed between the groups. LLC increased in the pneumonic group on the second day after the infection (p = 0.02) to return to baseline on the 5^th ^day. A moderate but not statistically significant increase was observed in the Pn15 group (p = 0.13).

**Table 2:** Lung liquid clearance (LLC) and lung wet to dry weight ratio (W/D). LLC increases on the second day post instillation and returns to baseline on the fifth day. W/D remains constant over time.

	W/D	LLC (%)
Ctr	4.33 ± 0.87	22.24 ± 3.65
*St*	4.29 ± 0.24	36.53 ± 4.95
*Pn 2*	4.66 ± 0.51	45.51 ± 4.26 *
*Pn 5*	4.03 ± 0.27	20.99 ± 5.94
*Pn 8*	3.47 ± 0.81	23.01 ± 2.80
*Pn 15*	3.92 ± 0.29	36.21 ± 8.23
*Pn 21*	4.31 ± 0.07	22.37 ± 2.56

*Footnote*: Data are mean (± SD). Comparisons between groups were made using analysis of variance with *post hoc *for multiple comparisons. *p < 0,05 vs the other groups.

**Ctr**: Control group, **St**: Sterile group, **Pn**: Pneumonic groups from the 2^nd ^to the 21^st ^days.

• Distal alveolar fluid clearance

**Figure 3:**
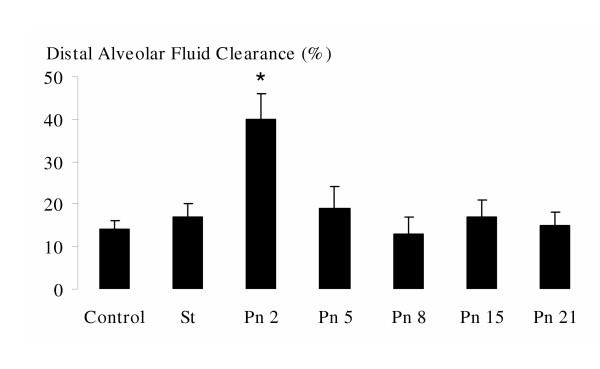
Evolution of the DAFC over time in sterile and infected beads injected groups. We observe an increase on the 2^nd ^day post infection, the clearance returns to a basal level on the 5^th ^day. *Footnote*: Data are mean (± SD). Comparisons between groups were made using analysis of variance with *post hoc *for multiple comparisons. *p < 0.05 vs the other groups. **DAFC**: distal alveolar fluid clearance, **Ctr**: Control group, **St**: sterile beads injected group, **Pn**: pneumonic groups from the 2^nd ^to the 21^st ^day.

Distal alveolar fluid clearance increased in the Pn2 group (Figure 3). This ratio decreased back to baseline on the 5^th ^day and remained comparable to both the St and the Ctr groups.

We tested whether the increase in DAFC observed at 48 hours was related to a TNF-α or a catecholamine dependent mechanism. No TNF-α was detected between the 2^nd ^and 21^st ^days in the serum or the alveolar compartment. Similarly, neither epinephrine nor nor-epinephrine could be detected in the alveolar compartment at 48 hours. The levels recovered in the plasma were comparable between control and pneumonic animals on the 2^nd ^and the 5^th ^days (Table 3).

**Table 3:** Plasma catecholamines measurement Plasma catecholamines were measured in pneumonic animals on the 2^nd ^and the 5^th ^day post instillation compared to the control group. No statistically significant difference could be observed.

	Ctr	Pn2	Pn5
*Epinephrine (μg/L)*	8.5 ± 2.1	11.2 ± 4.9	14.2 ± 3.5
*Norepinephrine (μg/L)*	5.8 ± 0.8	7.0 ± 2.2	8.2 ± 1.9

*Footnote*: Data are mean (± SD). Comparisons between groups were made using analysis of variance with *post hoc *for multiple comparisons. **Ctr**: Control group, **St**: Sterile group, Pn: Pneumonic groups.

### Distal airspace fluid clearance cannot be stimulated on the 5^th ^day post infection even after bacterial eradication

Even though DAFC returned to baseline values on the fifth day post infection, alveolar function was not normal in these chronically infected animals. First of all, since bacterial load persisted in the alveoli at least a modest increase of DAFC would have been expected in response to this stimulus. This absence of the expected response led us to test the DAFC response, in each group, to well known pharmacological stimuli.

• Terbutaline

**Figure 4:**
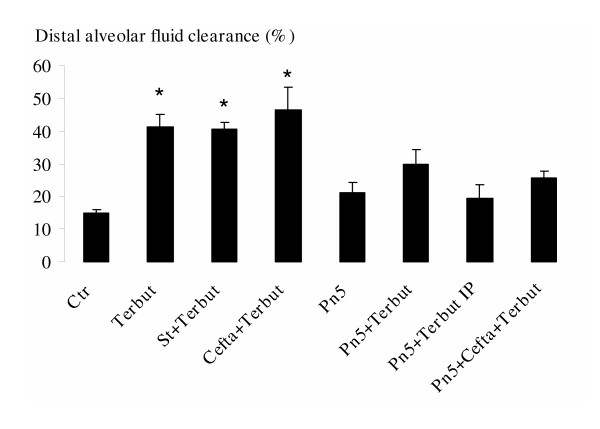
Evaluation of DAFC in the control compared to the pneumonic groups on the fifth day post instillation at baseline and after stimulation with terbutaline. A last group received terbutaline after bacterial eradication with ceftazidime administered intraperitoneally. None of the pneumonic groups could increase DAFC after either stimulation or bacterial eradication. *Footnote*: Data are mean (± SD). Comparisons between groups were made using analysis of variance with *post hoc *for multiple comparisons. *p < 0.05, statistically different from the control group. **DAFC**: distal alveolar fluid clearance. **Ctr**: Control group, **Terbut**: Control instilled with terbutaline (10^-4 ^M), **St + Terbut**: Sterile beads instilled with terbutaline (10^-4 ^M), **Cefta + Terbut**: Control group treated with ceftazidime (100 mg/kg/8 h for 72 h) and instilled with terbutaline (10^-4 ^M), **Pn5**: Pneumonic group on the 5^th ^day, **Pn5 + Terbut**: Pneumonic group on the 5^th ^day instilled with terbutaline (10^-4 ^M), **Pn5 + Terbut IP**: Pneumonic group on the 5^th ^day with an intraperitoneal injection of terbutaline, **Pn5 + Cefta+ Terbut**: Pneumonic group on the 5^th ^day treated with ceftazidime (100 mg/kg/8 h for 72 h) instilled with terbutaline (10^-4 ^M).

The administration of terbutaline is associated with an increase in DAFC in controls. Stimulation with terbutaline intratracheally could not increase DAFC on the 5^th ^day post infection, the intraperitoneal injection also failed to increase DAFC (Figure 4).

• Terbutaline after bacterial eradication

In order to eliminate the possibility of a direct bacterial effect inhibiting the expected response in the chronically infected animals, we performed a comparable stimulation with terbutaline on 10 animals treated with ceftazidime initiated 24 hours after the infection. On the 5^th ^day, all lungs were sterilized and measurement of ceftazidime levels showed a steady state level at 46.3 ± 4.8 μg/mL. However, even after the eradication of the pathogen, DAFC remained unresponsive to beta-adrenergic stimulation (Figure 4).

## Discussion

In our study we validated an experimental model allowing us to explore alveolar function in chronic *P. aeruginosa *lung infection through measurements of lung liquid movements. In this model of chronic *P. aeruginosa *lung infection, after observing an initial increase of both alveolar permeability and lung fluid movements, we characterized an impairment of DAFC where, even though DAFC returned to baseline, it remained unresponsive to pharmacological stimuli.

In the first part of our work, we validated, on several parameters, the chronic infection model previously described by Cash et al [8]. After reaching a peak on the second day of the infection and decreasing from the 5^th ^to the 15^th ^day, lung bacterial load persisted for 3 weeks. These results, as well as the analysis of the BAL and the histological features, are consistent with the literature [8,11,17,18].

Since, in this model, *P. aeruginosa *is associated with agar beads, we performed, as control groups, instillation of sterile agar beads. Sterile agar bead instilled rats did not show any evidence of weight loss and although they did present an increase in BAL cellularity, there were no PMN's except a slight increase on the second day which failed to reach a statistical significance (data not shown). This result is consistent with the literature, Nacucchio et al showed that agar beads alone could not reproduce the same level of injury than *P. aeruginosa *in agar beads [19].

From this first part of our work, we concluded that the model of chronic infection with *P. aeruginosa *is adequate, based on clinical, bacteriological, cytological and histological data.

Although a clinical study has reported increased lung permeability in COPD patients infected by *P. aeruginosa *[20], few studies have focused on the consequences of chronic lung infection on alveolar function and particularly fluid movements. In our study, lung fluid movements were maximal on the 2^nd ^day post infection. We observed an increase of alveolar-capillary barrier permeability, DAFC and overall lung liquid clearance. A normal lung wet to dry weight ratio was a consequence of this adequate alveolar response. This contrasts sharply with the data we obtained in an acute lung injury model where LLC dramatically decreased and W/D weight ratio increased at 4 and 24 hours after *Pseudomonas *instillation [7].

In our chronic model, following the increase in both permeability and lung liquid clearance, we observed an improvement in permeability with a return to baseline of these 2 parameters on the 5^th ^day.

The St group presented a moderate increase in permeability (Asp/plasma ratio: 0.31 ± 0.04), it has previously been reported that agar beads could alone be responsible for a moderate increase in permeability [19]. However, taking into account the association of the other parameters validating the model (clinical, bacteriological, cytological and histological), this effect does not challenge the model.

Our results showed an increase of the DAFC at 48 hours post infection. In acute lung injury, the initial alveolar response is usually towards an increase of DAFC which many authors have documented in septic shock [21], or after endotoxin administration [22]. In septic shock, this increase was related to the release of endogenous catecholamines. In acute *P. aeruginosa *pneumonia, increased DAFC can be related to either *Pseudomonas *exoproducts [15] or to a TNF-α dependent mechanism during the first 24 hours of the infection [4]. We tested in our model whether TNF-α or catecholamines could explain our results. TNF-α was not detectable and systemic endogenous epinephrine or nor-epinephrine not different from controls on the 2^nd ^or the 5^th ^day. TNF-α is produced during the early phase of pneumonia, and its short half life probably explains the absence of detectable levels at 48 hours. A dynamic evaluation of TNF-α production with serial samples or antibody neutralization experiments would be helpful to precisely study the role of TNF-α. We therefore did not rule out that TNF-α may have triggered an inflammatory response which could be responsible for the increased DAFC. Other potential mechanisms such as Transforming Growth Factor β remain to be investigated [23].

Surprisingly, on the fifth day, DAFC returned to baseline along with the improvement in permeability. Although it is logical to see an improvement in permeability, consistent with a decrease of the bacterial burden and an adequate host response, DAFC was expected to remain increased. The persisting presence of the pathogen in the alveoli and many factors only related to its presence would normally lead to a persistent increase of DAFC [15]. We therefore decided to evaluate if a normal increase in DAFC could be elicited on the 5^th ^day post infection in response to known pharmacological stimuli [24,25]. In the normal lung, intra-alveolar administration of terbutaline generates a DAFC increase of approximately 30% [26]. We observed comparable results in our study in control animals as well as animals which received only sterile beads. In our model, on the 5^th ^day, terbutaline intratrachéal administration did not change DAFC. However the lack of effect may be due to airway inflammation and an inability to adequately deliver the drug, we therefore decided to use intraperitoneal administration with the same agent. Our results also show the absence of DAFC increase. We then hypothesized that the absence of response to the stimulation might be related to the persistence of the pathogen in the alveoli. To test this hypothesis, we injected the animals with ceftazidime to sterilize the lungs on the 5^th ^day. Sterilization was achieved but failed to restore DAFC stimulation with terbutaline. To explain this impairment of DAFC, different hypotheses still remain to be investigated concerning these agonist's receptors and their regulation. Other authors have shown in different situations that either an internalization or a decrease of affinity of the receptors [27] could be observed. Another hypothesis could be a lost of sensitization through a decrease of the AMPc dependent signal transmission. It was shown, in vitro, on tracheal cells that a continuous or repeated exposure to isoproterenol could lead to a lost of sensitization [28].

If this unresponsiveness exists in patients, the absence of an adapted DAFC response in chronic lung infection could lead to major damage in the presence of any new lung injury. Although chronic lung infection has not been isolated, per se, as an aggravating factor associated to mortality in COPD patients admitted in an intensive care unit, a pre-existing underlying pathology is associated with a worsening of the prognosis in community and nosocomial pneumonia [29,30]. DAFC impairment might be part of the answer to this effect of underlying disease.

In conclusion, chronic *P. aeruginosa *pneumonia is characterized initially at 48 hours by an increased alveolar-capillary barrier permeability and an adapted host response with an increased DAFC and LLC preserving a normal lung wet to dry weight ratio. On the 5^th ^day, DAFC remains non responsive to pharmacological stimulation even after bacterial elimination. This impairment of DAFC could represent one of the factors responsible for the increased susceptibility of chronically infected patients to other respiratory insults.

## Authors' contributions

SB and FA were responsible for the acquisition of the data. KF and MOH made substantial contributions to the drafting of the manuscript and the analysis of the data. TP performed the radioactive labelling of the albumin (I^131^). EK was involved in the revision of the manuscript and the English editing. XL performed all the histological analysis. BG was involved in the acquisition of the data, the design and the conception of the study as well as the drafting of the article. All the authors read and approved the final manuscript.
